# Experiences of general practitioner continuity among women with chronic fatigue syndrome/myalgic encephalomyelitis: a cross-sectional study

**DOI:** 10.1186/s12913-016-1909-1

**Published:** 2016-11-14

**Authors:** Anne Helen Hansen, Olaug S. Lian

**Affiliations:** 1 Faculty of Health Sciences, Department of Community Medicine, University of Tromsø - The Arctic University of Norway, 9037 Tromsø, Norway; 2 University Hospital of North Norway, PO box 35, 9038 Tromsø, Norway

**Keywords:** Continuity of patient care, Chronic fatigue syndrome/myalgic encephalomyelitis (CFS/ME), Cross-sectional study, General practitioner, Primary health care, Norway

## Abstract

**Background:**

Continuity of care is important for patients with chronic illness in need of coordinated healthcare services from multiple providers. Little is known about how patients with chronic fatigue syndrome/myalgic encephalomyelitis (CFS/ME) experience continuity of GP care.

This study explores how women with CFS/ME experience GP care across the three dimensions of continuity: informational, management, and relational continuity.

**Methods:**

This cross-sectional study uses questionnaire data collected from members of The Norwegian ME Association. Descriptive statistics and logistic regressions were used to estimate experiences of continuity, and associations with age, education, self-rated degree of CFS/ME, duration of the GP relation (GP duration), and number of GP visits for CFS/ME-related issues during the previous year (GP frequency).

**Results:**

Almost two-thirds of participants reported positive experiences across all three dimensions of GP continuity of care; 64.4% for informational, 64.1% for management, and 77.2% for relational continuity. Lower educational attainment was associated with more negative experiences of informational continuity (primary school only compared to university educated: odds ratio [OR] 0.12, confidence interval [CI] 0.03–0.49, *p* = 0.003). Compared to participants aged 40–59 years, those aged 60+ years were significantly less likely to have experienced poor (negative) management continuity (OR 0.25, CI 0.09–0.76, *p* = 0.014). A GP relationship of three or more years was associated with positive experiences of relational continuity (OR 2.32, CI 1.09–4.95, *p* = 0.030). Compared to those with moderate CFS/ME, those who graded their CFS/ME as severe or very severe were significantly more likely to have negative experiences of relational continuity (OR 0.38, CI 0.14–0.99, *p* = 0.047).

**Conclusions:**

A large proportion of participants experienced all three aspects of continuity of GP care (especially the relational dimension) positively. Informational and management continuity scores were moderately lower. Our results suggest greater emphasis on information giving, feedback, and better coordination of care to be good strategies for practice improvement for this patient group.

**Electronic supplementary material:**

The online version of this article (doi:10.1186/s12913-016-1909-1) contains supplementary material, which is available to authorized users.

## Background

Patients with chronic fatigue syndrome/myalgic encephalomyelitis (CFS/ME) belong to the category of general practitioner’s (GP’s) patients who present with so-called medically unexplained symptoms. Their condition is characterised by a fluctuating long-term fatigue, persistent post-exertional malaise, sleep disturbances, pain, and other symptoms related to cognitive, immune, and autonomous dysfunction [1–3]. CFS/ME has a prevalence around 1–2 per thousand [4] and affects women more than men (70–85%) [5–7]. The terms CFS and ME are used interchangeably, and most often in combination with one another [1].

There are no diagnostic tests and no proven effective medical treatment for CFS/ME. The condition is medically contested, and challenges traditional distinctions between psyche and soma [8]. It poses particular problems to both patients and physicians regarding diagnosis, therapy, and communication [9]. CFS/ME patients often need healthcare services from multiple providers over a long period of time, and GPs are usually their key contact and care coordinator. As such, continuity of GP care is thought to be particularly important for this patient group. However, many GPs feel constrained by the scientific uncertainty of CFS/ME [10], not confident with diagnosing and treating the condition [11], and worry that the diagnostic label might be potentially harmful to patients [12]. Patients on the other hand, report feeling belittled, stigmatised, distrusted, rejected, and ignored by their doctors, and feel their moral character and the reality of their symptoms is being questioned [13, 14].

Continuity of care is a core value of general practice. Haggerty et al identify three types of continuity: Informational, management, and relational continuity [15]. Informational continuity relates to “the use of information on past events and personal circumstances to make current care appropriate for each individual” [15]. Management continuity is characterised by “a consistent and coherent approach to the management of a health condition that is responsive to a patient’s changing needs”, and relational continuity refers to “an ongoing therapeutic relationship between a patient and one or more providers” [15]. The authors argue that “for patients and their families, the experience of continuity is the perception that providers know what has happened before, that different providers agree on a management plan, and that a provider who knows them will care for them in the future” [15]. This paper explores experiences of these three aspects of continuity in general practice from a care seeker’s perspective.

All Norwegian citizens are provided a regular GP, and only 0.4% of the population choose to remain outside GPs’ lists [16]. This refers mostly to individual doctors (not group practices). Alongside universal tax-funding and gate-keeping, the list system provides strong incentives for continuity of individual GP care [17, 18]. Continuity is thought to increase patient satisfaction, compliance, and comprehensiveness of care [19–23]. In the drive for evidence-based improvements of health care delivery, research on care seekers’ experiences of continuity is important; especially among those with contested conditions and chronic diseases, who often require long-term care from multiple providers, typically coordinated by their GP. Little is known about how these patients experience continuity of GP care. Enhanced knowledge of their experiences might have important consequences for clinical practice, clinical outcomes, and for the planning and organising of health care services.

The aim of this study was to explore CFS/ME patients’ experiences of all three dimensions of continuity of GP care, to what extent participants considered these met/achieved by their primary care provider, and to what extent dimensions of continuity were associated with key participant clinical and socio-demographic, and GP-patient relationship, characteristics.

## Methods

### Data

This cross-sectional study used email survey data obtained in April/May 2013 from members of The Norwegian ME Association. Prior to distribution, the questionnaire was systematically tested [24] and piloted among 143 people belonging to the targeted group. Once finalised, invitations to participate were distributed by the Norwegian Social Science Data Service (NSD) Web Survey to a total of 811 ME Association members with known email addresses (about 40% of all members). The questionnaire included questions about demographic and socio-economic characteristics, health status in general, specific questions about symptoms, duration, severity, and treatment of CFS/ME, and use of and experiences with health care services. Participants experiences with health care services were predominantly measured using 4-point Likert scales. Non-respondents and those who had not completed filling in the questionnaire were given one reminder. Since we had no information about age or reasons for membership, members were asked to refrain from participating if they were below the age of 16 or did not suffer from CFS/ME themselves (health professionals, parents, others). We do not know how many of the non-respondents that were not eligible to participate, consequently an exact response rate cannot be calculated. Results from other parts of this dataset have previously been published [25, 26].

### Participants

**Fig. 1:**
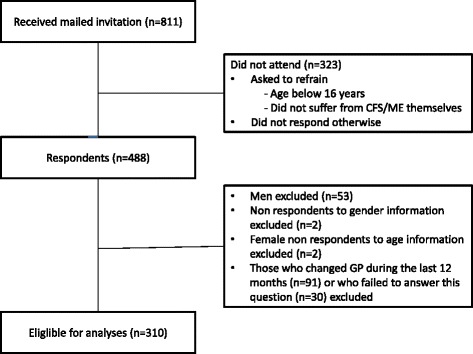
Flow chart of study participants

Women comprised 89.1% of the 488 respondents. Because of the gender distribution, as well as to avoid overfitting [27] and possible confounding effects of gender in the regression models, we excluded all men (53 respondents). We also excluded those who did not give gender and/or age information (4 respondents). Finally, since our aim was to study the patients’ experiences with their current GP, we excluded those who had changed GP during the last 12 months (*n* = 91) or who failed to answer this question (*n* = 30). This gives a net sample of 310 respondents (Fig. 1).

### Variables

Participants were informed of questions that only concerned their current GP and their CFS/ME symptoms over the previous 12 months. Dependent variables were based on the three questions presented in Table 1. The first question refers to informational continuity, the second to management continuity, and the third to relational continuity of care. For an easier interpretation of logistic regressions, all three variables were dichotomised by merging the original answering options presented in Table 1 into “not at all/ to a little extent” and “to some/a large extent”. Those who answered “not relevant” (the fifth answering option) were excluded from the analyses in the respective continuity category.

**Table 1:** Female CFS/ME patients’ experiences of continuity of GP care (%)

	To a large extent	To some extent	To a little extent	Not at all
	n (%)	n (%)	n (%)	n (%)
Has your GP given you feedback on investigations conducted by other health professionals? (*n* = 233) (informational continuity)	72 (30.9)	78 (33.5)	47 (20.2)	36 (15.4)
Does your GP fail to meet his/her tasks as a liaison between different parts of the health care services? (*n* = 226) (management continuity)	25 (11.1)	56 (24.8)	56 (24.8)	89 (39.3)
Do you experience that you and your GP are a well-functioning team? (*n* = 281) (relational continuity)	100 (35.6)	117 (41.6)	43 (15.3)	21 (7.5)

The independent variables were age, education, self-rated degree of CFS/ME, duration of the current GP relation (GP duration), and number of GP visits the previous year (GP frequency). Age was divided into three groups (Table 2). Six original education categories were merged into four due to low numbers in the outermost groups. Self-rated degree of CFS/ME was obtained from the question “What degree of ME do you have as of today?” As defined by an international consensus panel, four answering options were given: mild (about 50% reduction in activity), moderate (housebound most of the day), severe (bedridden most of the day), and very severe (completely bedridden) [28]. Due to low numbers, severe and very severe categories were merged. GP duration was obtained from the question “Approximately, how long have you had your current GP?” Responses were dichotomised into 0–2 years and 3 years or more. GP frequency was obtained from the question “Approximately how many times have you seen your GP, another GP or visited an emergency clinic during the previous 12 months for issues related to your ME?” The answers were categorised into 0 visits, 1–4 visits, 5–9 visits, and 10 visits or more.

**Table 2:** Sample characteristics

	Total sample	
	n	%
Age	310	100.0
16–39	87	28.1
40–59	177	57.1
60+	46	14.8
Education	301	100.0
Primary	23	7.7
High school	93	30.9
University 1–4 years	125	41.5
University 5 years +	60	19.9
Degree of CFS/ME	299	100.0
Mild	69	23.1
Moderate	199	66.6
Severe/very severe	31	10.3
GP duration	307	100.0
0–2 years	53	17.3
3 years+	254	82.7
GP frequency^a^	281	100
0 visits	25	8.9
1–4 visits	126	44.8
5–9 visits	84	29.9
10+ visits	46	16.4

^a^Number of GP visits the previous year for CFS/ME-related issues

### Analyses

Data were analysed using descriptive statistics and logistic regressions. Correlations were tested with Spearman’s rank correlation coefficients. We constructed three multivariable regression models, one for each of the dependent variables. The independent variables age, education, self-rated health, GP duration, and GP frequency were introduced collectively into all three models. For variables with three categories or more the largest group was designated the reference category. The dependent variables describing the patients’ experiences in the regression analyses were valued zero (0) for “not at all/ to a little extent”, and one (1) for “to some/a large extent” (Tables 3, 4 and 5).

**Table 3:** Female CFS/ME patients’ experiences of informational continuity from the question: “Have your GP given you feedback on investigations conducted by other health professionals?” (multivariable logistic regression)

	Feedback from the GP on investigations conducted by other health professionals to some or a large extent (*n* = 224)		
	OR	95% CI	*p*-value
Age			
16–39	0.76	0.38–1.53	0.444
40–59^a^	1.00	–	–
60+	1.49	0.60–3.71	0.392
Education			
Primary	**0.12**	**0.03–0.49**	**0.003**
High school	1.05	0.51–2.11	0.911
University 1–4 years^a^	1.00	–	–
University 5 years +	0.75	0.35–1.61	0.463
Self-rated degree of CFS/ME			
Mild	0.90	0.43–1.90	0.787
Moderate^a^	1.00	–	–
Severe/very severe	1.52	0.53–4.38	0.435
GP duration			
0–2 years^a^	1.00	–	–
3 years+	1.90	0.87–4.16	0.105
GP frequency^b^			
0 visits	0.37	0.11–1.22	0.103
1–4 visits^a^	1.00	–	–
5–9 visits	0.89	0.45–1.79	0.751
10+ visits	1.23	0.52–2.88	0.640

*GP* general practitioner, *OR* odds ratio, *CI* confidence interval

^a^Reference groups

^b^Number of GP visits the previous year for CFS/ME-related issues

Statistical significant findings (95% CI/*p* < 0.05) marked in bold

**Table 4:** Female CFS/ME patients’ experiences of management continuity from the question: “Does your GP fail to meet his/her tasks as a liaison between different parts of the health care services?” (multivariable logistic regression)

	GP fails to meet his/her tasks as a liaison between different parts of the health care services to some or a large extent (*n* = 217)		
	OR	95% CI	p-value
Age			
16–39	0.56	0.27–1.15	0.116
40–59^a^	1.00	–	–
60+	**0.25**	**0.09–0.76**	**0.014**
Education			
Primary	3.06	0.98–9.57	0.055
High school	1.32	0.63–2.77	0.471
University 1–4 years^a^	1.00	–	–
University 5 years +	1.94	0.88–4.31	0.102
Self-rated degree of CFS/ME			
Mild	0.60	0.27–1.32	0.201
Moderate^a^	1.00	–	–
Severe/very severe	1.19	0.46–3.09	0.720
GP duration			
0–2 years^a^	1.00	–	–
3 years+	0.93	0.42–2.04	0.854
GP frequency^b^			
0 visits	0.26	0.06–1.05	0.058
1–4 visits^a^	1.00	–	–
5–9 visits	0.50	0.24–1.03	0.060
10+ visits	0.44	0.18–1.04	0.060

*GP* general practitioner, *OR* odds ratio, *CI* confidence interval

^a^Reference groups

^b^Number of GP visits the previous year for CFS/ME-related issues

Statistical significant findings (95% CI/*p* < 0.05) marked in bold

**Table 5:** Female CFS/ME patients’ experiences of management continuity from the question: “Do you experience that you and your GP are a well-functioning team?” (multivariable logistic regression)

	The GP and the patient are a well-functioning team (*n* = 270)		
	OR	95% CI	*p*-value
Age			
16–39	1.30	0.63–2.71	0.471
40–59^a^	1.00	–	–
60+	0.90	0.36–2.24	0.816
Education			
Primary	0.56	0.19–1.69	0.306
High school	1.69	0.77–3.70	0.192
University 1–4 years^a^	1.00	–	–
University 5 years +	0.53	0.25–1.13	0.101
Self-rated degree of CFS/ME			
Mild	0.66	0.32–1.35	0.250
Moderate^a^	1.00	–	–
Severe/very severe	**0.38**	**0.14–0.99**	**0.047**
GP duration			
0–2 years^a^	1.00	–	–
3 years+	**2.32**	**1.09–4.95**	**0.030**
GP frequency^b^			
0 visits	0.75	0.27–2.10	0.590
1–4 visits^a^	1.00	–	–
5–9 visits	1.13	0.56–2.29	0.730
10+ visits	2.12	0.75–5.99	0.155

*GP* general practitioner, *OR* odds ratio, *CI* confidence interval

^a^Reference groups

^b^Number of GP visits the previous year for CFS/ME-related issues

Statistical significant findings (95% CI/*p* < 0.05) marked in bold

We used 95% confidence intervals (CI)/*p* < 0.05 as significance level throughout the study. All analyses were accomplished using Stata, version 14.0.

## Results

In total 488 members of the ME-association aged 16–73 years participated, constituting an overall response rate of approximately 60% (Fig. 1). Due to non-response from non-eligible receivers, and return of emails from email addresses not in use, the actual response rate is assumed to be higher. The 310 women eligible for analyses (Fig. 1) reported to have the diagnoses ME (*n* = 263), CFS (*n* = 26) and/or post viral fatigue syndrome (*n* = 50) (more than one diagnosis possible). Of these, three participants reported that a doctor had not diagnosed them, and one reported that she did not know if a doctor had given her the diagnosis.

The mean age of participants was 46.3 (CI 45.0–47.7) years. Most participants (61.3%) had suffered from CFS/ME for 10 years or more. The highest percentage of people were aged 40–59 years (57.1%), university educated (61.4%), had a moderate degree of CFS/ME (66.6%), had been with their GP for 3 years or more (82.7%), and had visited their GP 1–4 times during the previous year for CFS/ME related issues (44.8%) (Table 2). Overall, 91.1% had visited primary care at least once during the previous year for CFS/ME related issues (Table 2).

### Informational continuity

Nearly two-thirds (64.4%) of participants reported that their GP (to some, or a large extent) had provided feedback about the results of investigations conducted by other health professionals (e.g., secondary care specialists); however, over one-third (35.6%) of participants reported that this informational continuity had not been achieved. The multivariable analysis showed that participants with low educational attainment (primary school only), as opposed to those with higher educational attainment (university education of 1–4 years), were significantly more likely to have experienced poorer (negative) informational continuity (OR 0.12, CI 0.03–0.49, *p* = 0.003) (Table 3).

### Management continuity

Similarly, nearly two-thirds (64.1%) of participants reported that their GP (to some, or a large extent) had met his/her role as a liaison (coordinator) between different parts of the health services; however, over one-third (35.9%) of participants reported that management continuity was not achieved. Older people (aged 60+ years) were significantly more likely to have experienced positive management continuity from their GP compared to those aged 40–59 years (OR 0.25, CI 0.09–0.76, *p* = 0.014); meaning that there was a more positive attitude towards the GPs role as a liaison among older women (Table 4).

### Relational continuity

Nearly four in five (77.2%) of our participants considered themselves and their GPs (to some, or a large extent) as a well-functioning team; just over one-fifth (22.8%) of participants did not believe that relational continuity was provided (Table 1). The likelihood of experiencing themselves and their GP as a well-functioning team was reduced for those with severe/very severe CFS/ME compared to those with moderate CFS/ME (OR 0.38, CI 0.14–0.99, *p* = 0.047), whereas it was increased for those with a GP relation of 3 years or more compared to those with a shorter GP relationship (OR 2.32, CI 1.09–4.95, *p* = 0.030) (Table 5).

There were no strong correlations (defined as rho >0.5) between any of the independent variables in the regression models. C-statistics for the three models were 0.7718 (informational continuity), 0.8194 (management continuity), and 0.7840 (relational continuity).

## Discussion

### Summary

A larger proportion of CFS/ME patients experienced all three types of continuity more positively than negatively. Women in the lowest educational group were more likely to experience informational continuity negatively compared to women with a university education of 1–4 years. Women aged 60 years and over were more likely to experience management continuity positively compared to women aged 40–59 years. Relational continuity was more likely to be negatively experienced by women with severe disease, and more positively experienced by women with longer GP relationships (of 3 years or more).

### Comparison with existing literature

More than one in three participants had negative experiences with GPs feedback on investigations (informational continuity). This corresponds to the results of a British study where 40% of CFS patients reported that their test results were not explained by their GP [29]. Our finding that women with lower educational attainment were significantly more likely to experience negative informational continuity of GP care (Table 3) aligns with research showing that lower educated patients experience less information giving from doctors, fewer questions and discussions in consultations, and less decision making involvement compared to highly educated patients [30].

Almost two in three of our participants reported positive experiences of GP management continuity (GPs role as a liaison between health care services). Older women (aged 60+ years) were significantly more positive about management continuity than younger women were. This is consistent with the general finding that older patients are more satisfied with primary health care quality, where continuity is an important hallmark [31–33].

The most positive continuity of care score observed was for relational continuity. Nearly four in five (77.2%) of our participants considered themselves and their GP a well-functioning team. However, those who reported having more severe CFS/ME was significantly more likely to report this dimension of continuity as not met. This is consistent with a number of studies across diagnoses and health care settings, reporting that poorer health is associated with lower quality scores [34–38]. Unsurprisingly, a GP relationship of less than 3 years was associated with less positive relational continuity experiences. The duration of GP relationship might be considered an alternative measure of continuity [18], thus indicating that this association might represent two sides of the same coin.

### Strengths and limitations

One of the strengths of this study is that we recruited the participants from a relatively homogenous group (patient organisation). This recruitment strategy enabled patients to describe their experiences without fear of their views negatively affecting their relationships with health care providers. Moreover, we used a well-designed systematically tested questionnaire [24], and the estimated response rate is regarded to be high. This study contributes to fill gaps in our knowledge, since solid documentation of continuity of care experiences among patients with CFS/ME is lacking.

A limitation is that our sample may not be fully representative for women with CFS/ME. Higher functioning patients might not demand patient organisation membership to the same extent as those in poorer health (survivor bias) [39]. On the other hand, the most seriously affected members might not participate due to disease severity. The direction of a possible selection bias from these factors is not obvious. Second, the distribution of e-mail addresses might have been skewed, for instance towards younger members with higher education. However, since 93% of Norwegian households had access to the internet at the time of the study conduct [40] it is unlikely that this has influenced our results significantly. Third, our sample were younger and more highly educated than the Norwegian average [41]. Because younger individuals might not have completed their education, a possible skewness regarding these two variables might balance each other out to a certain extent.

In analysing data from questionnaires, there is always a potential for recall bias, usually leading to underreporting. Validity of self-reported measures might be discussed, particularly measures of disease severity. However, in the case of CFS/ME there are no objective measures for the presence or severity of disease. It is thus difficult to judge whether over-reporting or under-reporting might be present in our data.

Three participants reported that a doctor had not diagnosed them, and one reported that she did not know if a doctor had made her diagnosis. Since these few respondents might be waiting for further investigations as part of the diagnostic process, we do not think their inclusion represents a problem regarding the validity of our study. Besides, due to disagreement about diagnostic criteria and lack of objective tests for this condition, even doctor made diagnoses might be uncertain.

The possibility of unmeasured factors affecting the reported associations cannot be excluded. Further research on continuity of care among patients with CFS/ME might include exploring associations between continuity and other variables such as income and GP gender, and also examination of other facets of information giving, provision of feedback, doctor-patient collaboration, and coordination of healthcare services.

As with all cross-sectional studies, no causal relationships can be established.

## Conclusions

This study shows that a larger proportion of CFS/ME patients have positive rather than negative experiences of continuity of GP care. Informational and management continuity received the least positive scores, both with more than one in three participants expressing negative experiences. Nearly four in five regarded themselves and their GP as a well-functioning team. The results of our study indicate that although GPs are unable to offer an explanation or a quick fix therapy, simply because it does not exist, improvements of clinical practice are available within the field of communication. CFS/ME patients are in need of information, awareness, and understanding [42]. We therefore suggest an even stronger emphasis on information giving, provision of feedback, collaboration and coordination of healthcare services by GPs in the future. This might even influence the clinical outcome of CFS/ME [39] and lies within the skills and expertise that GPs’ have accumulated. Moreover, there is no significant risk associated with such a focus.

## Additional file

Additional file 1: Main sections covered in the questionnaire. (PDF 210 kb)

## Abbreviations

CFS/ME: Chronic fatigue syndrome/myalgic encephalomyelitis
CI: Confidence interval
GP: General practitioner
NSD: Norwegian social science data service
OR: Odds ratio

## References

[1] Brurberg KG, Fonhus MS, Larun L, Flottorp S, Malterud K (2014). Case definitions for chronic fatigue syndrome/myalgic encephalomyelitis (CFS/ME): a systematic review. BMJ open.

[2] Fukuda K, Straus SE, Hickie I, Sharpe MC, Dobbins JG, Komaroff A (1994). The chronic fatigue syndrome: a comprehensive approach to its definition and study. International Chronic Fatigue Syndrome Study Group. Ann Intern Med.

[3] Hairon N (2007). NICE guidance on managing chronic fatigue syndrome/ME. Nurs Times.

[4] Fluge O, Bruland O, Risa K, Storstein A, Kristoffersen EK, Sapkota D (2011). Benefit from B-lymphocyte depletion using the anti-CD20 antibody rituximab in chronic fatigue syndrome. A double-blind and placebo-controlled study. PLoS One.

[5] Bakken I, Tveito K, Gunnes N, Ghaderi S, Stoltenberg C, Trogstad L (2014). Two age peaks in the incidence of chronic fatigue syndrome/myalgic encephalomyelitis: a population-based registry study from Norway 2008 inverted question mark2012. BMC Med.

[6] Prins JB, van der Meer JW, Bleijenberg G (2006). Chronic fatigue syndrome. Lancet.

[7] Capelli E, Zola R, Lorusso L, Venturini L, Sardi F, Ricevuti G (2010). Chronic fatigue syndrome/myalgic encephalomyelitis: an update. Int J Immunopathol Pharmacol.

[8] Lian OS, Nettleton S. "United We Stand": Framing Myalgic Encephalomyelitis in a Virtual Symbolic Community. Qual Health Res. 2015;25(10):1383–94. 10.1177/1049732314562893 25488934

[9] Horton SM, Poland F, Kale S, Drachler Mde L, de Carvalho Leite JC (2010). Chronic fatigue syndrome/myalgic encephalomyelitis (CFS/ME) in adults: a qualitative study of perspectives from professional practice. BMC Fam Pract.

[10] Woodward RV, Broom DH, Legge DG (1995). Diagnosis in chronic illness: disabling or enabling--the case of chronic fatigue syndrome. J R Soc Med.

[11] Bowen J, Pheby D, Charlett A, McNulty C (2005). Chronic Fatigue Syndrome: a survey of GPs' attitudes and knowledge. Fam Pract.

[12] Chew-Graham C, Dowrick C, Wearden A, Richardson V, Peters S (2010). Making the diagnosis of Chronic Fatigue Syndrome/Myalgic Encephalitis in primary care: a qualitative study. BMC Fam Pract.

[13] Werner A, Malterud K (2003). It is hard work behaving as a credible patient: encounters between women with chronic pain and their doctors. Soc Sci Med.

[14] Anderson VR, Jason LA, Hlavaty LE, Porter N, Cudia J (2012). A review and meta-synthesis of qualitative studies on myalgic encephalomyelitis/chronic fatigue syndrome. Patient Educ Couns.

[15] Haggerty JL, Reid RJ, Freeman GK, Starfield BH, Adair CE, McKendry R (2003). Continuity of care: a multidisciplinary review. Bmj.

[16] Statistics about the GPs. [http://www.helsedirektoratet.no/finansiering/refusjonsordninger/tall-og-analyser/fastlege/Sider/statistikk-om-fastlegene.aspx] (in Norwegian). Accessed 11 Nov 2016.

[17] Norwegian Survey og Living Conditions 2008. [http://nsddata.nsd.uib.no/webview/index.jsp?study=http%3A%2F%2Fnsddata.nsd.uib.no%3A80%2Fobj%2FfStudy%2FNSD1327&studydoc=http%3A%2F%2Fnsddata.nsd.uib.no%3A80%2Fobj%2FfStudy%2FNSD1327&mode=documentation&] (in Norwegian). Accessed 11 Nov 2016.

[18] Hansen AH, Halvorsen PA, Aaraas IJ, Forde OH (2013). Continuity of GP care is related to reduced specialist healthcare use: a cross-sectional survey. Br J Gen Pract.

[19] Hjortdahl P, Laerum E (1992). Continuity of care in general practice: effect on patient satisfaction. Bmj.

[20] Aboulghate A, Abel G, Elliott MN, Parker RA, Campbell J, Lyratzopoulos G (2012). Do English patients want continuity of care, and do they receive it?. Br J Gen Pract.

[21] O'Connor PJ, Desai J, Rush WA, Cherney LM, Solberg LI, Bishop DB (1998). Is having a regular provider of diabetes care related to intensity of care and glycemic control?. J Fam Pract.

[22] Saultz JW, Lochner J (2005). Interpersonal continuity of care and care outcomes: a critical review. Ann Fam Med.

[23] Cabana MD, Jee SH (2004). Does continuity of care improve patient outcomes?. J Fam Pract.

[24] Lian OS, Wilsgaard T (2004). Patient satisfaction with primary health care before and after the introduction of a list patient system. Tidsskr Nor Laegeforen.

[25] Lian OS, Hansen AH. Factors facilitating patient satisfaction among women with medically unexplained long-term fatigue: A relational perspective. Health (London, England: 1997) 2015. 10.1177/1363459315583158 25979224

[26] Hansen AH, Lian OS (2016). How do women with chronic fatigue syndrome/myalgic encephalomyelitis rate quality and coordination of healthcare services? A cross-sectional study. BMJ Open.

[27] Babyak MA (2004). What you see may not be what you get: a brief, nontechnical introduction to overfitting in regression-type models. Psychosom Med.

[28] Carruthers BM, van de Sande MI, De Meirleir KL, Klimas NG, Broderick G, Mitchell T (2011). Myalgic encephalomyelitis: International Consensus Criteria. J Intern Med.

[29] Deale A, Wessely S (2001). Patients' perceptions of medical care in chronic fatigue syndrome. Soc Sci Med.

[30] Aelbrecht K, Rimondini M, Bensing J, Moretti F, Willems S, Mazzi M (2015). Quality of doctor-patient communication through the eyes of the patient: variation according to the patient's educational level. Adv Health Sci Educ Theory Pract.

[31] Raivio R, Jaaskelainen J, Holmberg-Marttila D, Mattila KJ (2014). Decreasing trends in patient satisfaction, accessibility and continuity of care in Finnish primary health care - a 14-year follow-up questionnaire study. BMC Fam Pract.

[32] Ali FM, Nikoloski Z, Reka H. Satisfaction and responsiveness with health-care services in Qatar-evidence from a survey. Health Policy. 2015;119(11):1499–505. 10.1016/j.healthpol.2015.09.012 26511059

[33] Van Den Assem B, Dulewicz V (2015). Doctors' trustworthiness, practice orientation, performance and patient satisfaction. Int J Health Care Qual Assur.

[34] Trentman TL, Chang YH, Chien JJ, Rosenfeld DM, Gorlin AW, Seamans DP (2014). Attributes associated with patient perceived outcome in an academic chronic pain clinic. Pain Pract.

[35] Poot AJ, den Elzen WP, Blom JW, Gussekloo J (2014). Level of satisfaction of older persons with their general practitioner and practice: role of complexity of health problems. PLoS One.

[36] Hekkert KD, Cihangir S, Kleefstra SM, van den Berg B, Kool RB (2009). Patient satisfaction revisited: a multilevel approach. Soc Sci Med.

[37] Doyle C, Lennox L, Bell D. A systematic review of evidence on the links between patient experience and clinical safety and effectiveness. BMJ open 2013, 3(1). doi: 10.1136/bmjopen-2012-001570. 10.1136/bmjopen-2012-001570 PMC3549241 23293244

[38] Heje HN, Vedsted P, Sokolowski I, Olesen F (2008). Patient characteristics associated with differences in patients' evaluation of their general practitioner. BMC Health Serv Res.

[39] Gladwell PW, Pheby D, Rodriguez T, Poland F (2014). Use of an online survey to explore positive and negative outcomes of rehabilitation for people with CFS/ME. Disabil Rehabil.

[40] Bruk av IKT i husholdningene, 2012, 2. kvartal [Use of ICT in households, 2012, 2nd quarter] [ssb.no/teknologi-og-innovasjon/statistikker/ikthus/aar/2016-09-06] (in Norwegian). Accessed 11 Nov 2016.

[41] Population’s level of education, 1 October 2015 [www.ssb.no/en/utdanning/statistikker/utniv/aar/2016-06-20] (in Norwegian). Accessed 11 Nov 2016.

[42] Comiskey C, Larkan F (2010). A national cross-sectional survey of diagnosed sufferers of myalgic encephalomyelitis/chronic fatigue syndrome: pathways to diagnosis, changes in quality of life and service priorities. Ir J Med Sci.

