# Supplementary material for: Experiences of general practitioner continuity among women with chronic fatigue syndrome/myalgic encephalomyelitis: a cross-sectional study
Source: BMC Health Serv Res. 2016 Nov 14;16:650. doi: 10.1186/s12913-016-1909-1 (PMC5109710; doi:10.1186/s12913-016-1909-1)
Supplement: Additional file 1: — Main sections covered in the questionnaire. (PDF 210 kb) [file 12913_2016_1909_MOESM1_ESM.pdf]

Attachment:

Questions and answers in the survey that are used in the current paper

**A. Background information:**
**Age** (open-ended)
**Education**<br><br>- Primary<br><br>- High school<br><br>- University 1-4 years<br><br>- University 5 years +
**Degree of CFS/ME**<br><br>- Mild<br><br>- Moderate<br><br>- Severe<br><br>- Very severe
**GP duration** (open-ended)
**GP frequency** (open-ended)
**B. Experiences from consultations for CFS/ME-related issues with current GP:**
Has your GP given you feedback on investigations conducted by other health professionals? (informational continuity)<br><br>- to a large extent<br>- to some extent<br>- to a little extent<br>- not at all

Does your GP fail to meet his/her tasks as a liaison between different parts of the health care services? (management continuity)<br>- to a large extent<br>- to some extent<br>- to a little extent<br>- not at all
Do you experience that you and your GP are a well-functioning team? (relational continuity)<br>- to a large extent<br>- to some extent<br>- to a little extent<br>- not at all

In addition to these questions, the questionnaire contained the following topics:

1) Other background information: age of illness onset and time from illness onset to diagnosis; income etc.

2) GP gender, changes of regular GP, reasons for not having contact with current GP

3) Questions about advice received from GPs, degree of compliance and experieces of treatment options

4) Experiences from contacts with health services shortly after illness onset

5) Overall satisfacation with Norwegian national health services

6) Degree of satisfaction with current GP in relation to aspects such as being trusted, believed and taken seriously; having a shared understanding of the ailment, etc.
